# A Retrospective Audit of the Management of Shoulder Dislocations at a Trauma Unit

**DOI:** 10.7759/cureus.87256

**Published:** 2025-07-03

**Authors:** Maham Mansoor, Sunandan Datta, Bratati Bandyopadhyay, Rachala Madhu, Muhammad Tahir Raza, Mohammed Wahaaj Hussain, Mustafa Al-Jaafar

**Affiliations:** 1 Trauma and Orthopaedics, Aneurin Bevan University Health Board, Newport, GBR; 2 Trauma and Orthopaedics, The Grange University Hospital, Cwmbran, GBR

**Keywords:** audit, orthopaedic emergency, posterior dislocation of the shoulder, shoulder anterior dislocation, shoulder instability, upper limb injuries

## Abstract

Introduction: Shoulder dislocation is the most common major joint dislocation encountered in our emergency department, with anterior dislocation accounting for the vast majority. This retrospective audit evaluates adherence to the health board guidelines for managing traumatic shoulder instability at Grange University Hospital.

Methods: Data from 100 patients presenting with native shoulder dislocations between January 2021 and February 2024 were analyzed. Clinical notes, radiological records, and procedural details were reviewed to assess compliance with imaging protocols, reduction procedures, and follow-up care.

Results: The mean patient age was 44 years, with a female predominance of 52% (52 out of 100). Anterior dislocations constituted 83% of cases (83 patients), with posterior and inferior dislocations accounting for 16% (16 patients) and 1% (one patient), respectively. Manipulation under anaesthesia (MUA) was successful in 98% of cases (98 out of 100 patients), while 2% (two patients) required open reduction. Pre- and post-reduction imaging was performed in 97% of patients. Computed tomography (CT) and magnetic resonance imaging (MRI) were frequently used to assess associated injuries, which included Hill-Sachs lesions, greater tuberosity fractures, and rotator cuff tears. Neurological complications were identified in 28 patients (28%), primarily involving the axillary nerve. Posterior dislocations were more prevalent than typically reported, suggesting heightened diagnostic awareness.

Conclusion: This audit demonstrates strong compliance with established clinical protocols, reflected in high MUA success rates and effective identification of injury-related complications. However, areas for improvement have been identified to further improve clinical management and patient satisfaction.

## Introduction

Shoulder dislocations are the most frequently encountered major joint dislocations in clinical practice, accounting for nearly 50% of all large joint dislocations [1]. This high incidence is primarily attributed to the anatomical configuration of the glenohumeral joint, which features a shallow glenoid fossa and a highly mobile humeral head, a construct that inherently predisposes the joint to instability, particularly among young, physically active individuals. Among these, anterior dislocations are the most prevalent, comprising over 90% of all shoulder dislocation cases [2,3], while posterior dislocations account for approximately 2-4% [4].

Epidemiologically, shoulder dislocations are more common in males, with nearly 50% occurring between the ages of 15 and 29. A second peak in incidence is observed in elderly females during the ninth decade of life [5].

Anterior dislocations typically result from a fall on an outstretched hand, whereas posterior dislocations are more often associated with tonic-clonic seizures, electrical injuries, or direct anterior trauma to the shoulder. While anterior dislocations generally present acutely, posterior dislocations often have a more insidious onset and are classified as acute only if diagnosed within three weeks of the inciting event [5,6].

It is essential to diagnose shoulder dislocations early, as delay in reduction and detection of concomitant rotator cuff tears can significantly worsen functional outcomes in the elderly, as observed by Wang [7]. In young patients at risk of recurrent instability (with risk factors such as young age, male sex, participation in contact sports, or shoulder hypermobility), prompt detection and manipulation, combined with the appropriate use of magnetic resonance imaging (MRI) scans, can aid in formulating effective management strategies. Therefore, this study aims to assess how our current clinical practice aligns with established standards of care worldwide [7,8].

The aim of this retrospective audit was to evaluate adherence to our health board's clinical pathway for managing traumatic anterior shoulder instability. This pathway aligns closely with the guidelines established by the British Orthopaedic Association (BOA) and the British Elbow and Shoulder Society (BESS) [9,10].

## Materials and methods

This retrospective audit was conducted at The Grange University Hospital (GUH), the acute care hospital within our health board. The study period spanned from January 2021 to February 2024.

According to our institutional protocol, all patients presenting to the Emergency Department (ED) with native shoulder joint dislocations undergo a comprehensive clinical assessment, including radiographic imaging. Standard imaging includes anteroposterior and axillary or Grashey (glenoid profile) views. The Grashey view is particularly valuable for identifying Bankart and Hill-Sachs lesions.

Following imaging, manipulation under anaesthesia (MUA) is attempted by ED physicians or the on-call trauma and orthopaedics team. Post-manipulation, neurovascular status is assessed and documented. Patients then undergo post-reduction radiographs while immobilized in a polysling or shoulder immobilizer. If reduction is confirmed, patients are typically discharged after a short admission and followed up in a specialist upper limb clinic. Additional imaging, if required, is arranged during outpatient follow-up by consultant specialists.

Patients requiring further intervention are scheduled for MUA or open reduction under fluoroscopy in either the emergency or the next available trauma theatre.

Inclusion and exclusion criteria

The study included all patients presenting to the ED of GUH with native shoulder joint dislocations within the defined study timeframe. Patients who experienced an episode of shoulder dislocation while an inpatient at any of the hospital wards were excluded from the study. Patients who presented to the ED with a shoulder dislocation and then absconded were also excluded.

Data collection

Data were collected from January 2021 to February 2024, encompassing 100 patients. The inclusion criteria included all patients presenting to GUH with native shoulder dislocations that required MUA or surgical intervention. Exclusion criteria included prosthetic shoulder dislocations and chronic dislocations presenting at elective sites.

Data sources included the hospital's electronic systems: Ormis, Clinical Workstation (CWS), and Synapse (for radiological review). Clinical notes, operative records, and clinic letters were thoroughly analyzed.

Reduction techniques

Various closed reduction techniques for anterior shoulder dislocations were employed, including Kocher's method, Matsen's technique, and the Milch technique. These methods, which involve traction-countertraction and/or leverage, were selected based on clinician familiarity and have comparable outcomes [11,12].

Posterior dislocations were managed with gentle in-line traction of the internally rotated and adducted arm, combined with anterior manipulation of the humeral head. Countertraction using an axillary sheet was applied when necessary [13].

Advanced statistical analysis was not conducted, as the audit's primary focus was on assessing guideline compliance rather than establishing statistical associations. The study aimed to evaluate clinical practice patterns and identify areas for improvement, making descriptive analysis sufficient for addressing the audit objectives within the context of quality improvement.

## Results

Patient demographics

A total of 100 patients presented to our ED with a shoulder dislocation during the study duration (Table 1). The mean age at presentation was 44 years (range: 14-100 years; interquartile range (IQR): 31 years). Our study revealed that 21 patients (21%) were in their seventh decade, 23 patients (23%) were in their eighth decade, and eight patients (8%) were under 30 years old. Gender distribution was quite symmetrical, with 48 male patients (48%) and 52 female patients (52%).

**Table 1:** Demographics of patients presenting with native shoulder dislocations

Demographics	Results, n	Results, %
Number of shoulder dislocations	100	100
Male patients	48	48
Female patients	52	52

Imaging modalities and timing

Our audit revealed that 97 out of 100 (97%) patients underwent radiography both before and after MUA, whereas three out of 100 (3%) had them only after MUA. Out of 100, 32 patients (32%) needed a computed tomography (CT) scan, 31 (31%) required an MRI scan, two patients (2%) underwent ultrasonography (USG), one (1%) had a CT arthrogram, and three (3%) needed magnetic resonance (MR) arthrogram.

Direction of dislocation

**Figure 1:**
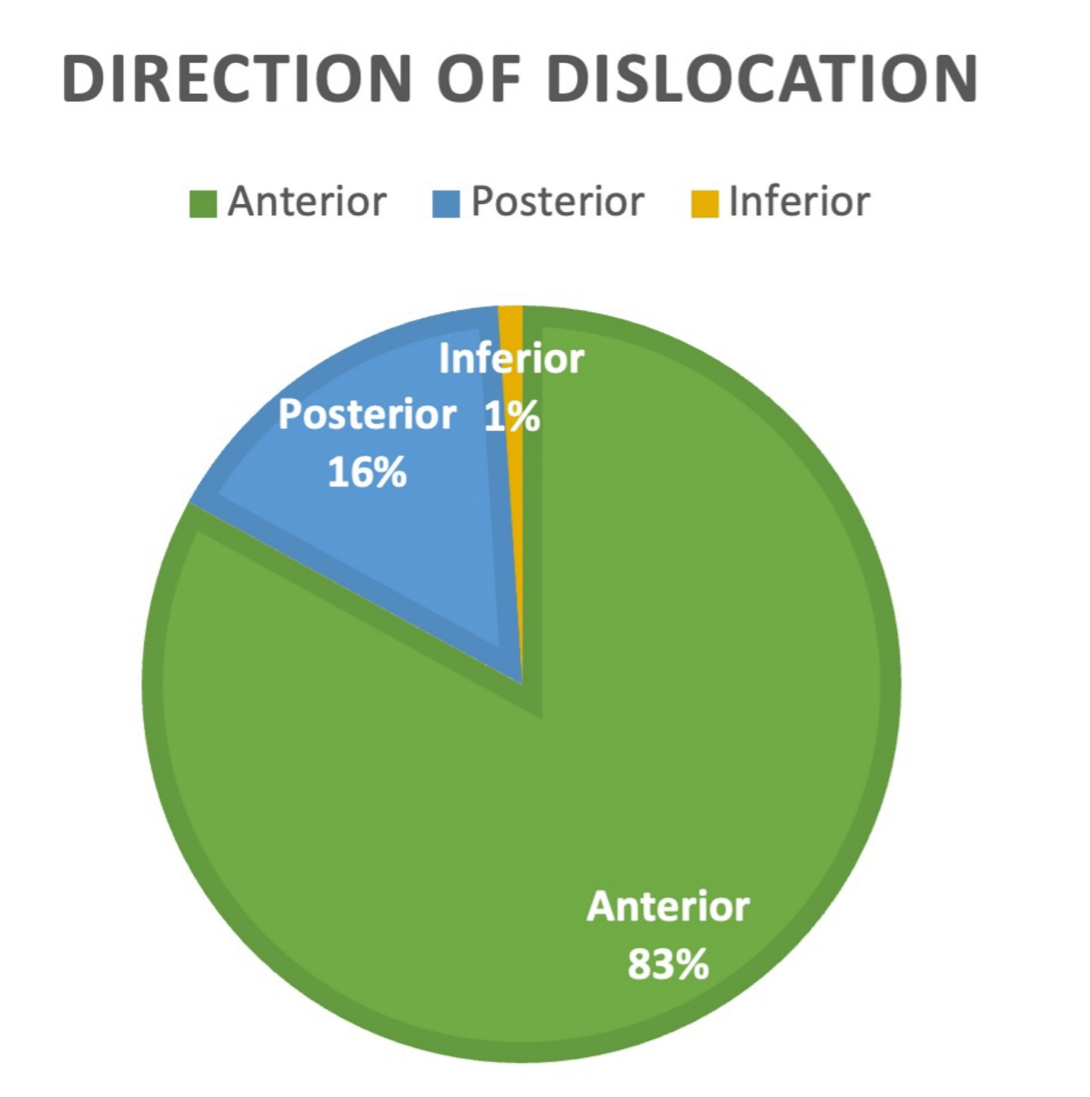
Direction of shoulder dislocation among patients The majority of dislocations were anterior (83%), followed by posterior (16%) and inferior (1%) (N = 100).

An overwhelming 83 out of 100 (83%) patients sustained an anterior dislocation, whereas 16 out of 100 (16%) patients sustained a posterior dislocation, and only one patient (1%) had an inferior dislocation. This is represented in Figure 1.

Primary procedure

In 98 out of 100 patients (98%), MUA was successful in reducing the dislocated shoulders; however, two patients (2%) required open reduction and definitive surgery for reduction of the dislocated shoulder.

Timing, number of manipulations, and location

The mean time to reduction after presentation was 152 minutes. The location of reduction was the ED in 88 cases (89.8%) and the trauma theatre in 10 cases (10.2%). The time to reduction was 109 minutes for those manipulated in the ED and 217 minutes for those manipulated in the theatre. A total of 82 patients (82%) were successfully reduced after one manipulation attempt, 15 required two attempts, and only three patients (who required manipulation or open reduction in theatre) needed three attempts.

Outcomes after MUA and surgeries

A total of 57 patients (57%) underwent physiotherapy alone after their MUA, and three patients (3%) underwent repeat manipulation in theatres, as they had repeat dislocations before being discharged. Sixteen patients (16%) underwent open reduction and internal fixation (ORIF) for concomitant proximal humerus (greater tubercle) fractures. Four patients (4%) underwent arthroscopic rotator cuff repair. Three patients (3%) underwent shoulder hemiarthroplasty, five underwent reverse shoulder arthroplasty, and two were treated with the modified Latarjet procedure. Ten patients were lost to follow-up.

Associated pathologies

**Figure 2:**
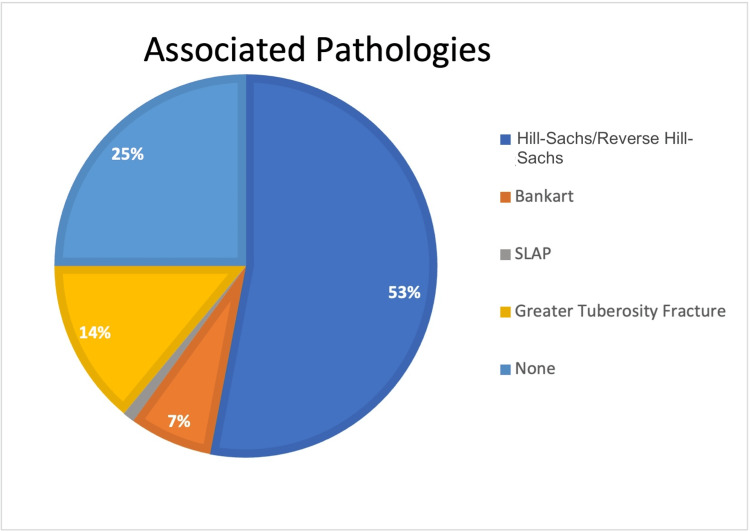
Proportion of patients having associated pathological lesions (N = 100) SLAP: superior labrum anterior posterior

Our audit revealed that out of the 100 patients, 29 (29%) had accompanying Hill-Sachs and reverse Hill-Sachs lesions, 16 had greater tuberosity fractures, eight (8%) had Bankart lesions, and one (1%) had superior labrum anterior posterior (SLAP) lesions. This is elucidated in Figure 2.

A total of 14 patients (14%) had radiological findings consistent with a rotator cuff injury; 14 (14%) had no such injury, and eight (8%) had no comment on rotator cuff morphology. It is important to note that comments on rotator cuff morphology were only applicable to patients who underwent an MRI scan, an MR arthrogram, or a US scan.

Complications

Table 2 illustrates the details of the complications recorded after manipulation of traumatic shoulder dislocations.

**Table 2:** Patients having complications associated with shoulder dislocation

Complication	Number, n	Percentage, %
Nerve injury	28	28
Recurrent dislocation	7	7
Limited range of motion	23	23
Frozen shoulder	3	1
Non-union	1	1

**Figure 3:**
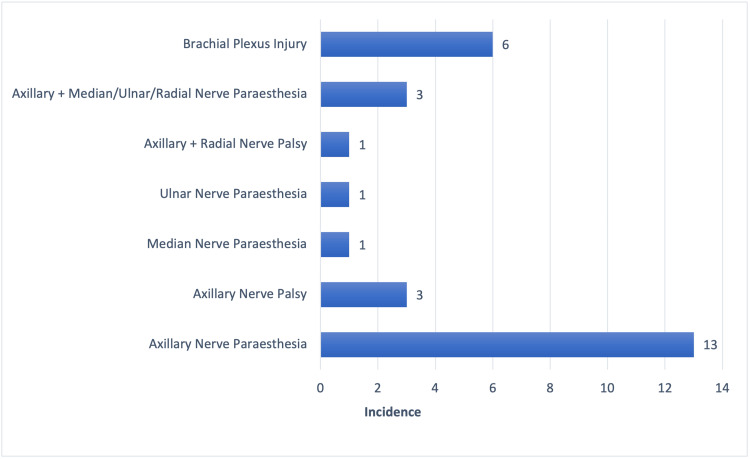
Numbered bar diagram showing neurological injuries associated with shoulder dislocation (N = 100)

Among those patients with nerve injuries, axillary nerve involvement was the most common, followed by brachial plexus injuries (Figure 3).

## Discussion

This retrospective audit provides a comprehensive overview of adherence to local guidelines based on the BOA/BESS standards for the management of shoulder dislocations at our health board. The data reflect consistent application of protocol-driven care in the acute management of shoulder dislocations at GUH.

The predominance of anterior shoulder dislocations (83%) aligns with established literature, which reports that over 90% of shoulder dislocations are anterior in nature [2,3]. Notably, our cohort included a higher proportion of elderly patients, particularly those in their seventh and eighth decades of life. This contrasts with previous studies, which have highlighted a peak incidence in younger males aged 15-29 years [5]. The observed variation may reflect local population demographics or the increased incidence of falls and age-related bone fragility in older adults. It is also well-documented that shoulder dislocations in the elderly are more frequently associated with concomitant injuries such as rotator cuff tears or proximal humerus fractures. Additionally, older patients are more likely to present with neurovascular injuries, particularly involving the axillary nerve or brachial plexus [14].

Our results are consistent with those of Robinson et al., who reported that 33.4% of patients with traumatic anterior shoulder dislocation sustained either rotator cuff tears or greater tuberosity fractures. In our study, a similar proportion (30%) demonstrated these associated injuries [15].

Imaging guidelines were generally well adhered to, with 97% of patients receiving both pre- and post-reduction radiographs, in line with best-practice protocols. The remaining 3%, who received only post-reduction imaging, may reflect exceptional clinical circumstances; however, this merits further review to ensure no systemic lapses exist. The frequent use of CT (33%) and MRI (34%) indicates a proactive approach in identifying associated pathology. These advanced imaging modalities frequently revealed additional findings such as Hill-Sachs lesions, proximal humerus fractures, and rotator cuff injuries [16,17].

Neurological complications were identified in 28% of patients, consistent with the wide range reported in existing literature (0.4%-65.5%). The axillary nerve was the most commonly affected, followed by the brachial plexus. Prior studies have reported axillary nerve involvement in approximately 10% of anterior dislocations. Most brachial plexus injuries in our cohort were postganglionic, infraclavicular, and continuous, typically presenting as neuropraxia or axonotmesis, both of which generally have a favourable prognosis [18-20].

Closed reduction under MUA had a high success rate, with 98% of dislocations successfully managed without the need for operative intervention. Only two patients required open reduction, underscoring the effectiveness of current non-operative management strategies when supported by adequate analgesia and sedation. Neurovascular assessments were consistently documented pre- and post-reduction, demonstrating strong adherence to safety protocols.

A notable finding in our audit was the relatively high incidence of posterior shoulder dislocations (16%) compared to the expected range of 2%-4% [4]. Posterior dislocations are frequently associated with reverse Hill-Sachs lesions, potentially explaining their higher detection rate in this audit. This discrepancy may indicate either heightened diagnostic vigilance or an unexpectedly greater local prevalence. Given the often subtle presentation of posterior dislocations, this finding underscores the importance of comprehensive radiographic evaluation and heightened clinical suspicion [21].

This audit has identified areas for improvement in service delivery. Not all patients under 40 years of age received an MR arthrogram, despite this being the recommended imaging modality for evaluating soft tissue injuries in younger individuals [22]. While a large proportion of our cohort underwent CT, stricter adherence to imaging guidelines is warranted to avoid missing concomitant fractures that may not be evident on plain radiographs. This is particularly important given the increased prevalence of glenoid or humeral bone loss in the elderly population [23].

Despite the comprehensive nature of this audit, several limitations exist. As a single-site, retrospective study with a relatively modest sample size, the generalizability of our findings to other institutions with different demographics or clinical protocols may be limited. Additionally, 10 patients were lost to follow-up, which may have impacted the accuracy of outcome assessments. The absence of standardized functional outcome scores further limits our ability to evaluate long-term clinical effectiveness. Nonetheless, our findings remain consistent with previous evidence [19,21].

## Conclusions

This audit demonstrates that management of shoulder dislocations at our institution aligns closely with local and national guidelines, with high rates of successful MUA, consistent use of imaging, and effective identification of associated pathologies. The findings reinforce the importance of standardized protocols in achieving good clinical outcomes and highlight areas such as radiographic documentation and post-reduction assessment where further improvement is possible. Areas for improvement were identified, particularly regarding the underuse of MR arthrograms in younger patients and occasional omission of post-reduction radiographs. Future audits could benefit from evaluating longer-term functional outcomes and recurrence rates, particularly in elderly and high-risk populations.
